# Complete Genome Sequence of *Spiroplasma helicoides* TABS-2^T^ (DSM 22551), a Bacterium Isolated from a Horsefly (*Tabanus abactor*)

**DOI:** 10.1128/genomeA.01201-16

**Published:** 2016-10-20

**Authors:** Wei-Yi Shen, Wen-Sui Lo, Yi-Ching Lai, Chih-Horng Kuo

**Affiliations:** a Institute of Plant and Microbial Biology, Academia Sinica, Taipei, Taiwan; b Molecular and Biological Agricultural Sciences Program, Taiwan International Graduate Program, National Chung Hsing University and Academia Sinica, Taipei, Taiwan; c Graduate Institute of Biotechnology, National Chung Hsing University, Taichung, Taiwan; d Biotechnology Center, National Chung Hsing University, Taichung, Taiwan

## Abstract

*Spiroplasma helicoides* TABS-2^T^ (DSM 22551) was isolated from the gut of a horsefly (*Tabanus abactor*) collected near Ardmore, Oklahoma, USA, in 1987. Here, we report the complete genome sequence of this bacterium to facilitate the investigation of its biology and the comparative genomics among *Spiroplasma* species.

## GENOME ANNOUNCEMENT

*Spiroplasma helicoides* is a horsefly-associated bacterium found in North America (1). The type strain TABS-2^T^ was isolated from the gut of a horsefly (*Tabanus abactor*) collected near Ardmore, Oklahoma, USA, in 1987 and was assigned to group XXXII within the genus. Biochemical characterization of this strain demonstrated that it is capable of catabolizing glucose but does not hydrolyze arginine or urea (1). To facilitate future investigation on the biology of this bacterium, as well as to improve the taxon sampling of available *Spiroplasma* sequences for comparative and evolutionary studies (2), we determined the complete genome sequence of *S. helicoides* TABS-2^T^.

The strain was acquired from the German Collection of Microorganisms and Cell Cultures (catalogue number DSM 22551). The freeze-dried sample was processed according to the manufacturer’s instructions and cultured in M1D medium (3) prior to DNA extraction using the Wizard Genomic DNA purification kit (Promega, USA). PCR and Sanger sequencing was performed to verify that the 16S rRNA gene sequence matched the reference record (GenBank accession no. NR_025704.1).

The procedures for sequencing, assembly, and annotation were based on those described in our previous studies on *Spiroplasma* genomes (4–13). Briefly, the Illumina MiSeq platform was used to generate 300-bp reads from one paired-end library (~550-bp insert, 2,327,640 reads) and one mate-pair library (~4,200-bp insert, 2,828,554 reads). The initial *de novo* assembly was performed using ALLPATHS-LG release 52188 (14). Subsequently, PAGIT version 1 (15) was used to assist an iterative process for improving the assembly. For each iteration, the raw reads were mapped to the assembly using BWA version 0.7.12 (16), programmatically checked using the MPILEUP program in SAMtools package version 1.2 (17), and visually inspected using IGV version 2.3.57 (18). Polymorphic sites and gaps were corrected based on the mapped reads. The process was repeated until the complete genome sequence was obtained. The programs RNAmmer (19), tRNAscan-SE (20), and Prodigal (21) were used for gene prediction. The gene names and product descriptions were first annotated based on the homologous genes in other *Spiroplasma* genomes (4–13) as identified by OrthoMCL (22). Subsequent manual curation was based on BLASTp (23) searches against the NCBI nonredundant database (24) and the KEGG database (25, 26). Putative clustered regularly interspaced short palindromic repeats (CRISPRs) were identified using CRISPRFinder (27).

The circular chromosome of *S. helicoides* TABS-2^T^ is 1,326,546 bp in size and has a G+C content of 26.8%; no plasmid was found. The first version of annotation includes one set of 16S-23S-5S rRNA genes, 29 tRNA genes (covering all 20 amino acids), 1,151 protein-coding genes, four pseudogenes, and one CRISPR locus (chromosomal positions 1,068,189 to 1,071,129; containing 44 spacers).

### Accession number(s).

The complete genome sequence of *S. helicoides* TABS-2^T^ has been deposited at DDBJ/EMBL/GenBank under the accession number CP017015.
